# Interactions between C-Reactive Protein Genotypes with Markers of Nutritional Status in Relation to Inflammation

**DOI:** 10.3390/nu6115034

**Published:** 2014-11-11

**Authors:** Cornelie Nienaber-Rousseau, Bianca Swanepoel, Robin C. Dolman, Marlien Pieters, Karin R. Conradie, G. Wayne Towers

**Affiliations:** Centre of Excellence for Nutrition, North-West University, Private Bag x6001, Nutrition, Box 594, Potchefstroom 2520, South Africa; E-Mails: 20546025@nwu.ac.za (B.S.); robin.dolman@nwu.ac.za (R.C.D.); marlien.pieters@nwu.ac.za (M.P.); karin.conradie@nwu.ac.za (K.R.C.); wayne.towers@nwu.ac.za (G.W.T.)

**Keywords:** CRP, diet-gene interactions, inflammation therapy, nutrigenetics, systemic inflammation

## Abstract

Inflammation, as indicated by C-reactive protein concentrations (CRP), is a risk factor for chronic diseases. Both genetic and environmental factors affect susceptibility to inflammation. As dietary interventions can influence inflammatory status, we hypothesized that dietary effects could be influenced by interactions with single nucleotide polymorphisms (SNPs) in the *CRP* gene. We determined 12 *CRP* SNPs, as well as various nutrition status markers in 2010 black South Africans and analyzed their effect on CRP. Interactions were observed for several genotypes with obesity in determining CRP. Lipid intake modulated the pro-inflammatory effects of some SNPs, *i.e.*, an increase in both saturated fatty acid and monounsaturated fatty acid intake in those homozygous for the polymorphic allele at rs2808630 was associated with a larger increase in CRP. Those harboring the minor alleles at rs3093058 and rs3093062 presented with significantly higher CRP in the presence of increased triglyceride or cholesterol intake. When harboring the minor allele of these SNPs, a high omega-6 to -3 ratio was, however, found to be anti-inflammatory. Carbohydrate intake also modulated *CRP* SNPs, as HbA1C and fasting glucose levels interacted with some SNPs to influence the CRP. This investigation highlights the impact that nutritional status can have on reducing the inherent genetic susceptibility to a heightened systemic inflammatory state.

## 1. Introduction

Circulating C-reactive protein (CRP) is an important non-specific, systemic inflammatory biomarker that represents the action of numerous activated cytokines. CRP is associated with various disease states [1,2,3,4], such as cardiovascular disease (CVD) [5,6]. Treatments aimed at reducing systemic inflammation may therefore reduce morbidity and mortality and are highly relevant for public health.

Inflammation is a modifiable risk factor, which is amenable to lifestyle interventions involving diet and exercise [7], concomitant with weight regulation [8,9,10]. Strong anti-inflammatory dietary influences have been determined for foods with a decreased glycemic index (GI) and load, as well as in individuals with an increased intake of fiber, magnesium, vitamin E (alpha-tocopherol), carotenoids and flavonoids [11,12,13]. The traditional Mediterranean dietary pattern, which is characterized by a high ratio of monounsaturated fatty acids (MUFA) to saturated fatty acids (SFA), a high omega-3 to -6 polyunsaturated fatty acids (PUFA) ratio and high intakes of fruit, vegetables, legumes and grains, has also been determined to decrease inflammation [12,13,14]. Increased intake of SFA, total fatty acids and high-GI carbohydrates, as well as a high omega-6 to -3 ratio, on the other hand, is associated with a pro-inflammatory state [11,12,13].

Apart from lifestyle factors, CRP concentrations are also influenced by certain genetic variations, such as single nucleotide polymorphisms (SNPs) within the *CRP* gene at 1q21-q23 [15,16]. The etiology of the multifactorial diseases associated with CRP concentrations, mentioned previously, is intimately tied to both lifestyle factors and the genetic make-up of an individual, and it is important to consider both when investigating these disorders. Although both dietary factors and genetic susceptibility have major effects on a person’s inflammatory state, very few investigations have been undertaken into their interactive effects. We hypothesize that the anti-inflammatory effects of dietary interventions are due to the interactive effects these components have on genetic susceptibility to inflammation inherent in an individual. We therefore investigated whether there are interactions between markers of nutritional status (anthropometrical markers, biochemical markers and dietary components that were previously associated with inflammation), and *CRP* gene SNPs on CRP concentrations. This may increase our understanding of the manner in which nutritional status modulates genetic susceptibility to inflammation to provide knowledge to predict the therapeutic success of lifestyle interventions better.

## 2. Experimental Section

### 2.1. Study Design, Population Selection and Demographic Characteristics

This is a cross-sectional study nested within the South African arm of the Prospective Urban and Rural Epidemiology (PURE) study [17]. Participants were selected in a three-stage sampling process based on power calculations. Communities were selected, after which households were randomly chosen within the communities. The head of each selected household provided voluntary, written informed consent before completing an interviewer-based questionnaire. The questionnaire was used to screen for potentially eligible individuals within the households, *i.e.*, free-living Africans aged 35 to 70 years, with no overt diseases, no reported usage of chronic medication and/or any self-reported acute illness.

### 2.2. Ethics

Those who met the inclusion criteria were informed of all aspects of the study and provided written informed consent prior to participation. Participants had the option of withdrawing from the study at any time. The collected data was treated confidentially by performing analyses on coded data. The study protocol was approved by the Ethics Committee of the North West University (NWU-00016-10-A1) and adhered to the guidelines laid down in the Declaration of Helsinki of 1975, revised in 2000.

### 2.3. Lifestyle Factors and Dietary Intake Assessment and Analysis

Information on demographic and lifestyle factors, including tobacco use, was obtained from an interviewer-based questionnaire. A questionnaire (developed by Vaz *et al.* [18]) was used to determine physical activity (PA). The PA index, developed and tested in the Transition in Health during Urbanization in South Africa (THUSA) study, was used in combination with a step counter to determine PA.

Dietary intakes were ascertained with a culturally sensitive validated quantitative food frequency questionnaire (QFFQ) developed in South Africa [19,20]. Participants were interviewed by trained fieldworkers in the participants’ language of choice, to recall their usual food intake (food and beverages) by reporting the frequency, amount and preparation of the food consumed over the previous month. Portion sizes were estimated using visual aids including food portion photographs [21], food models, household measures and various food labels. The QFFQ data was computerized using the FoodFinder3^®^ program (Medical Research Council, Tygerberg, South Africa) and sent to the Medical Research Council of South Africa for computerization, verification and nutrient analysis based on the most recent South African Food Composition Tables. Two predefined diet quality scores, the Healthy Diet Indicator and Adapted Thiele dietary quality score, were adapted and calculated, as previously described [22].

### 2.4. Anthropometrical and Blood Pressure Assessment

The body mass and height of participants, wearing minimal clothing, were measured to calculate the body mass index (BMI; kg/m^2^). Upper-arm, waist and hip circumferences were measured at the level of the mid-acromiale-radiale landmarks, the narrowest waist and the greatest protrusion of the buttocks, respectively. The waist-to-hip ratio was calculated. Systolic and diastolic blood pressures (blood pressure cuff on left arm) were measured with the OMRON HEM-757 apparatus (Omron Healthcare, Kyoto, Japan).

### 2.5. Blood Sampling and Storage

Fasting blood samples were drawn. Whole blood was collected in citrate, ethylenediamine tetra-acetic acid and fluoride tubes and were centrifuged at 2000× *g* for 15 min at room temperature to yield buffy coat for deoxyribonucleic acid (DNA) extraction and 15 min at 2000× *g* at 10 °C to yield plasma for glycated hemoglobin A1c (HbA1c) and glucose measurements, respectively. Coagulated blood was centrifuged at 2000× *g* at 10 °C for 15 min to yield serum for lipid and CRP analysis. Aliquots were snap frozen before storage at 70 °C until analysis.

### 2.6. Biochemical Analyses

Serum high sensitivity CRP and lipid concentrations (*i*.*e*., total cholesterol (TC), high density lipoprotein cholesterol (HDL-C) and triglycerides) were measured using a Sequential Multiple Analyzer Computer (Konelab 20i, Thermo Fisher Scientific Oy, Vantaa, Finland). Low density lipoprotein cholesterol (LDL-C), was calculated using the Friedewald formula [23]. Plasma glucose and HbA1c concentrations were determined via a hexokinase method using the Synchron^®^ Systems (Beckman Coulter Co., Fulleron, CA, USA) and with the D-10 Hemoglobin testing system (Bio-Rad Laboratories, Hercules, CA, USA), respectively. Human immunodeficiency virus (HIV) status was determined according to the protocol of the South African Department of Health, using whole blood for the rapid first response HIV Card Test 1-2.0 (Transnational Technologies Inc., PMC Medical, Nani Daman, India) and the outcome was confirmed with a Pareeshak test (BHAT Bio-tech, Bangalore, India). Appropriate pre- and post-test counseling was provided to the participants.

Genomic DNA was extracted using the FlexiGene™ kit (QIAGEN Inc., Valencia, CA, USA; catalogue number 51206) and Maxwell^®^ 16 blood DNA purification kit (Promega Corporation, Madison, WI, USA). The latter was used when insufficient yield was obtained from the first method, as established by the NanoDrop™ spectrophotometer (ND-1000, Wilmington, DE, USA). The *CRP* genes of a sub-sample of 30 randomly selected participants from the PURE-SA study were sequenced using the ABI Prism^®^, BIGDye^®^ Terminator version 3 Ready Reaction Cycle Sequencing Kit (Applied Biosystems, Foster City, CA, USA). The resultant electropherograms were aligned according to the reference *CRP* gene (Genbank accession AF449713) using BioEdit (version 7.1.3.0, Ibis Biosciences, Carlsbad, CA, USA) and were used to identify alterations. An *in silico* search for *CRP* SNPs that affect CRP concentrations was performed to identify the observed alterations [24]. Prior to the genotyping of the identified variants using the Illumina^®^ VeraCode GoldenGate assay technology on a BeadXpress^®^ platform (Illumina^®^ Inc., San Diego, CA, USA), SNPs were scored (varying from 0 to 1) by the Assay Design Tool to determine the viability of each specific assay. Only bi-allelic SNPs could be measured using this technique. Genotyping was performed for 12 *CRP* SNPs. For quality control purposes, SNPs with a gencall score of >0.5 and a call rate ≥0.9 were included in the analysis.

The expected genotype frequencies were calculated according to the assumptions of adhering to Hardy-Weinberg equilibrium (HWE) and compared to those observed in our study by the chi square (χ^2^) test [25]. Haploview software (developed in Mark Daly’s laboratory at the Massachusetts Institute of Technology) [26] was used to calculate the level of pairwise linkage-disequilibrium (LD) between the *CRP* SNPs using both D’ and *r*^2^ values [27].

### 2.7. Statistical Analysis

The computer software package Statistica^®^ version 12 (Statsoft Inc., Tulsa, OK, USA) was used for the statistical analyses. The distributions of all of the variables were skewed. Descriptive statistics were calculated and continuous variables were presented as median (interquartile range). Differences in CRP concentrations between the genotypic subgroups were analyzed using the Kruskal-Wallis analyses of variance.

Participants infected with HIV were asymptomatic and as the CRP concentrations did not differ between infected and uninfected individuals (*p* = 0.22), infected ones were not excluded from the analysis. Spearman correlations were performed to test for statistical dependence between variables.

Further analyses were performed on individuals for whom both genotypic and CRP concentration data (*n* = 1588) was available. In the data reported here, individuals with high CRP concentrations were not excluded, as doing so resulted in bias against individuals carrying the alleles responsible for an increase in inflammation; however, we determined that exclusion of those with high CRP (>10 mg/L) did not materially affect our results. To investigate whether different factors (anthropometry, biochemical variables, dietary components) have interactive effects with the various *CRP* polymorphisms in predicting CRP concentrations, analyses of covariance (ANCOVA), were performed. To account for multiple testing, the method of Hochberg and Benjamini [28] was used. Only interactions that remained after adjusting for multiple testing and after excluding possible statistical outliers were reported as significant.

## 3. Results

### 3.1. Characteristics of Study Participants and Correlations between Measured Variables and CRP Concentrations

The characteristics of the participants, including data on several dietary intake factors that have been reported to have modulating effects on inflammation, are presented in Table 1. For subjects with CRP data 461 had metabolic syndrome and 874 were hypertensive. Spearman correlations revealed that age, anthropometric markers, lipid profile variables, HbA1c and PA levels correlated significantly (*p* < 0.05) with CRP and these were therefore treated as possible confounding factors in the subsequent ANCOVAs. Other variables also correlated significantly with CRP, but had weak correlations (*r* < 0.2) that could be ascribed to the large sample size [29].

**Table 1:** Characteristics of the study participants.

Participant’s Characteristics			Whole Group (*n* = 2010)	Correlation with CRP	
				*r*	*p*
Age (year)			48.0 (41.0–56.0)	0.11	<0.001
Blood pressure (mmHg)	Diastolic		87.0 (78.0–97.0)	0.07	<0.001
	Systolic		130 (116–147)	0.05	0.03
Tobacco use (%)	Current		52.7	0.02	0.30
	Former		4.14		
	Never		42.7		
Physical activity			7.33 (5.79–8.68)	−0.11	<0.001
Anthropo-metrical markers	BMI (kg/m^2^)		22.9 (19.3–28.6)	0.22	<0.001
	Waist circumference (cm)		77.5 (70.2–87.7)	0.27	<0.001
	Hip circumference (cm)		93.1 (84.8–106)	0.20	<0.001
	Waist-to-hip ratio		0.83 (0.78–0.88)	0.09	<0.001
	Upper-arm circumference (cm)		27.5 (24.3–31.3)	0.20	<0.001
Biochemical markers	HIV status (*n*) (−/+/do not know)		1668/326/14	0.03	0.14
	TC (mmol/L)		4.82 (4.01–5.87)	0.03	0.21
	LDL-C (mmol/L)		2.79 (2.08–3.65)	0.07	0.004
	HDL-C (mmol/L)		1.42 (1.06–1.87)	−0.14	<0.001
	Triglycerides (mmol/L)		1.08 (0.82–1.55)	0.13	<0.001
	Fasting glucose (mmol/L)		4.80 (4.30–5.30)	0.07	0.003
	HbA1c (%)		5.50 (5.30–5.80)	0.21	<0.001
	CRP (mg/L)		3.29 (0.96–9.34)		
Dietary intake	Energy (KJ)		7175 (5268–10001)	0.05	0.05
	Protein (%TE)		11.6 (10.4–12.9)	0.01	0.60
	Carbohydrate (%TE)		60.3 (54.2–67.5)	−0.01	0.57
	Added sugar (%TCHO)		15.2 (9.51–21.8)	0.05	0.05
	Total fat (%TE)		22.5 (17.4 – 27.7)	0.03	0.23
	SFA (%TE)		5.29 (3.63–7.06)	0.03	0.17
	MUFA (%TE)		5.77 (3.75–7.74)	0.05	0.05
	MUFA-to-SFA ratio (g:g)		1.13 (0.97–1.27)	0.03	0.24
	PUFA	PUFA (%TE)	6.78 (5.06–8.60)	0.03	0.2
		Omega-6 (g)	12 (6.98–18.5)	0.003	0.91
		Omega-3 (g)	0.34 (0.20–0.52)	0.004	0.88
		Omega-6 to -3 ratio (g:g)	36.1 (26.1–50.3)	0.005	0.83
	Cholesterol (mg)		150 (80.0–259)	0.07	0.002
	Alcohol intake (g/day)		11.5 ± 22.7	−0.01	0.58
	Abstainers:drinkers (*n*:*n*)		824:659		
	Dietary fiber (g)	Total	19.1 (13.7–27.0)	0.02	0.29
		Insoluble	2.02 (1.15–3.31)	0.08	<0.001
		Soluble	1.46 (0.84–2.44)	0.08	<0.001
	Vitamin C (mg)		17.7 (10.4–38.1)	0.07	0.003
	Vitamin E (mg)		9.67 (5.92–14.2)	0.03	0.26
	Mg (mg)		285 (199–406)	0.03	0.20
	Zinc (mg)		8.90 (6.49–12.6)	0.03	0.15
	Carotenoids (μg)		1117 (303–2305)	0.09	<0.001
	Fruit and vegetables (g)		86.6 (51.9–154)	0.08	<0.001
Pulses, nuts and seeds (g)			4.29 (0–19.3)	0.02	0.49
Adapted Thiele dietary quality score			1490 (1275–1630)	0.07	0.003
Healthy diet indicator			6.68 (6.11–7.21)	0.03	0.14

Numbers are slightly different for several variables owing to missing data. No differences in CRP concentrations were detected between subgroups of tobacco use. Continuous data was presented as median (interquartile range).

This article focuses on the combined effect of several anthropometric, biochemical and dietary components with genetics in relation to inflammation. Detailed results of the individual effects of these SNPs on CRP concentrations are described elsewhere [30]. A summary of the effects of the 12 SNPs on CRP concentrations is provided in Table 2. All genotyped SNP frequencies adhered to the assumption of HWE and were within the 95% confidence interval ranges. Of the SNPs investigated, rs3093058 with rs3093062 and rs2027471 with rs1341665 were not independent of each other. Three genetic variations (rs3093058, rs3093062, and rs303068) were associated with a significant (*p* < 0.05) increase, while five (rs1205, rs1341665, rs2794520, rs7553007 and rs2027471) were associated with a decrease in CRP concentrations.

**Table 2:** Summary of the minor allele frequencies of the investigated *CRP* genotypes and their effects on CRP concentrations.

*CRP* Gene Polymorphisms	Genotype	MAF%	ΔCRP
rs3093058 (−790A > T)	AA	T = 19.4	Increase
	AT		
	TT		
rs1205 (3′ UTR) (3u2131C > T or 3872A > G or +2147G > A)	GG	A = 22.8	Decrease
	GA		
	AA		
rs7553007	GG	A = 23.8	Decrease
	GA		
	AA		
rs2794520	GG	A = 23.6	Decrease
	GA		
	AA		
rs2808630 (+5237A > G)	AA	G = 14.3	No change
	AG		
	GG		
rs3093068 (+2911C > G)	CC	G = 37.5	Increase
	CG		
	GG		
rs1417938 (intron) (i178T > A)	TT	A = 97.5	No change
	TA		
rs3093062 (−409G > A)	GG	A = 16.2	Increase
	GA		
	AA		
rs2027471	TT	A = 23.5	Decrease
	TA		
	AA		
rs1130864 (3′ UTR) * (3u1273C > T or +1444C > T)	GG	A = 13.4	No change
	GA		
	AA		
rs1341665 * (−7180C > T)	GG	A = 23.5	Decrease
	GA		
	AA		
rs1800947 (exon 2) (L184L or +1059C > G)	CC	C = 100	No change
	CG		

CRP concentrations are reported as least square means adjusted for confounders (95% confidence intervals). * Where alleles differ between the gene names, the difference is due to reference to the antisense strand. A, adenine; C, cytosine; CRP, C-reactive protein; G, guanine; MAF, minor allele frequency; rs, reference sequence; T, thymine.

### 3.2. Interaction Effects of Various Markers of Nutritional Status with CRP SNPs in Relation to the CRP Phenotype

#### 3.2.1. PA, Anthropometry and *CRP* Genotype Interactions in Relation to Inflammation

**Figure 1:**
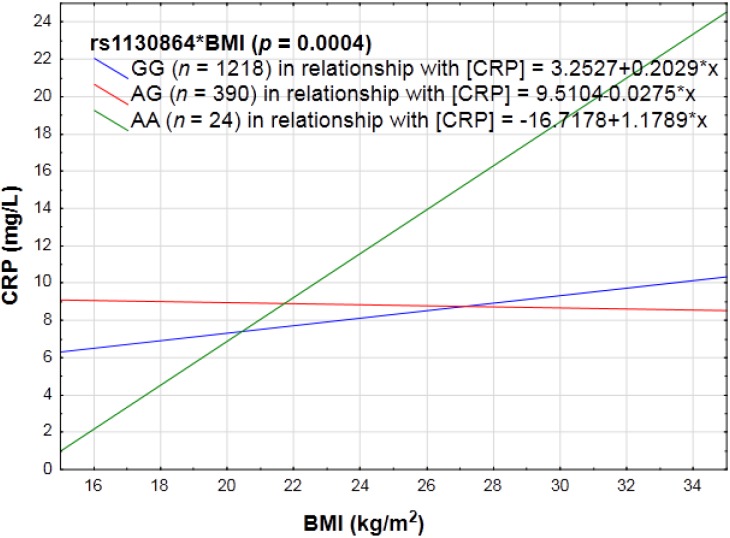
Example portraying the linear relationship between an anthropometrical variable, body mass index (BMI), with CRP concentrations categorized for rs1130864.

Factorial ANCOVAs indicated that BMI and upper-arm circumference interacted with the variant at rs1130864 (*p* = 0.004; *p* = 0.003), thus altering its effect on the CRP phenotype. When homozygous for the variant allele (A) at this locus, it was determined that the higher the BMI or arm circumference, the higher the CRP concentrations (see Figure 1). With the addition of the major allele (G) the rate of change in CRP became less pronounced.

Waist circumference, which is a crude indicator of abdominal fat, influenced CRP concentrations together with rs3093058 (*p* = 0.04) and rs3093062 (*p* = 0.04). CRP concentrations increased as waist girth increased, but with every addition of the minor allele at rs3093058 and rs3093062 the effect became more pronounced. No interactions were observed for hip circumference or waist-to-hip ratio. PA did not interact with any of the measured *CRP* SNPs in influencing inflammation.

#### 3.2.2. Biochemical Markers of the Lipid Profile, as Well as Glycemic Control (HbA1c and Fasting Glucose) and *CRP* Genotype Interactions in Relation to Inflammation

**Figure 2:**
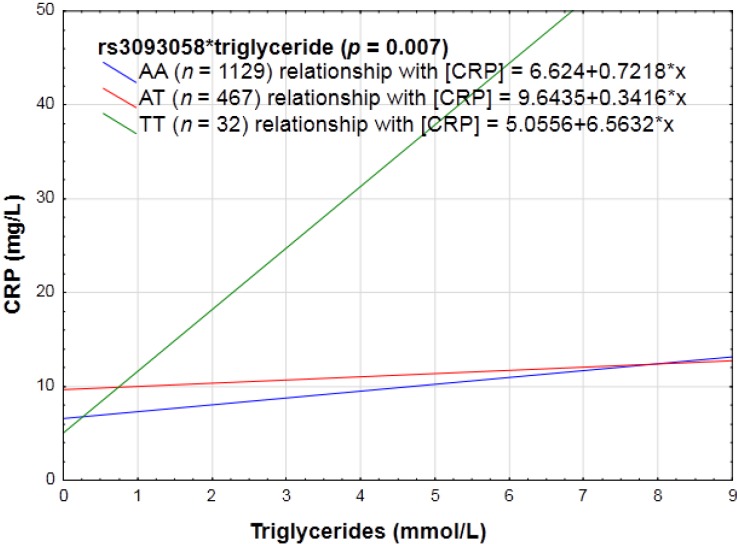
The linear relationship between triglycerides in relation to CRP concentrations categorized for rs3093058.

TC, LDL-C or HDL-C concentrations did not interact with any of the SNPs to influence CRP concentrations. Triglyceride concentrations interacted with rs3093058 (*p* = 0.007), rs3093062 (*p* = 0.007), and rs1130864 (*p* = 0.03), thus influencing the CRP phenotype. The interactions with rs3093058 and rs3093062 were the same, *i.e.*, when homozygous for the polymorphic allele, CRP increased significantly more than when harboring the major allele (see Figure 2). When harboring the minor allele at rs1130864, increasing triglyceride concentrations did not promote inflammation, but when homozygous for the major allele increasing triglyceride concentrations were pro-inflammatory.

**Figure 3:**
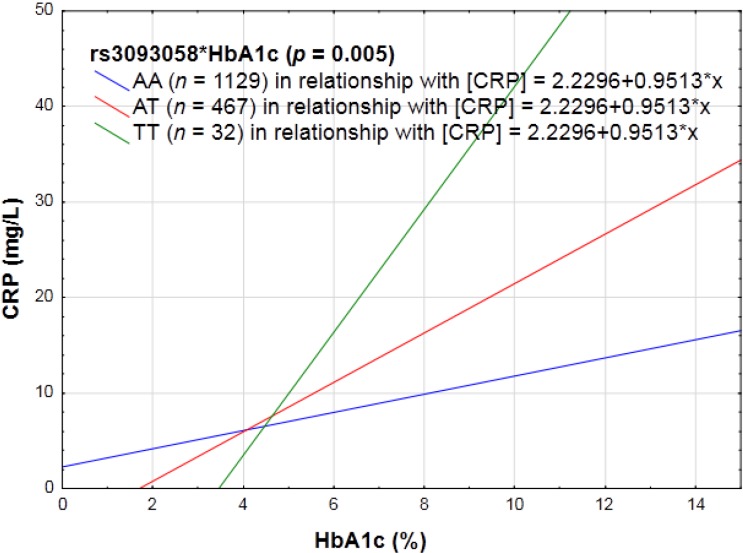
The linear relationship between HbA1c in relation to CRP concentrations categorized for rs3093058.

HbA1c changed the way in which rs3093058 (*p* = 0.005), rs3093068 (*p* = 0.006) and rs3093062 (*p* = 0.004) influenced the CRP phenotype. CRP concentrations were significantly increased in a genotype dose-dependent manner with each addition of the minor allele at these loci (see Figure 3).

**Figure 4:**
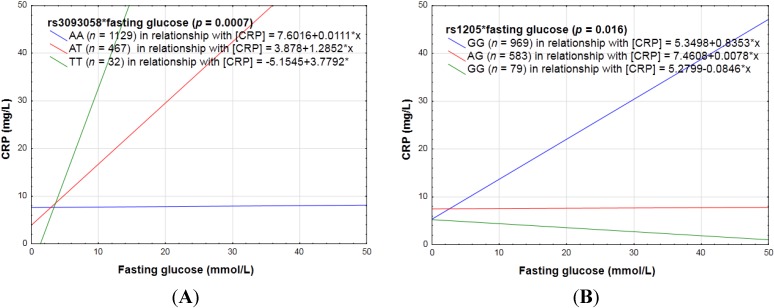
Examples portraying the linear relationship between fasting glucose in relation to CRP concentrations categorized for *CRP* SNPs with (**A**) a pro- (**B**) or anti-inflammatory effect with the addition of the polymorphic allele.

Interaction effects also existed for fasting glucose; it changed the way in which the following SNPs affected CRP concentrations: rs3093058 (*p* = 0.0007), rs1205 (*p* = 0.016), rs7553007 (*p* = 0.02), rs2794520 (*p* = 0.019), rs2808630 (*p* = 0.018), rs3093068 (*p* = 0.001), rs3093062 (*p* = 0.0004), rs2027471 (*p* = 0.02), and rs1341665 (*p* = 0.02). The minor alleles at rs3093058, rs3093068 and rs3093062 were pro-inflammatory, while at the other loci the minor alleles were anti-inflammatory in a genotype dose-dependent manner (see Figure 4).

#### 3.2.3. Dietary Sugar Intake, Fat Intake, Absolute Alcohol Intake, Dietary Fiber, Vitamin C and E, Magnesium, Zinc, Carotenoid, Fruit and Vegetables, Pulses, Nuts and Seeds, as Well as Diet Quality and *CRP* Genotype Interactions in Relation to Inflammation

Carbohydrate, or added sugar intake expressed as a percentage (%) of total energy (TE), had no interactions with any of the *CRP* genotypes.

**Figure 5:**
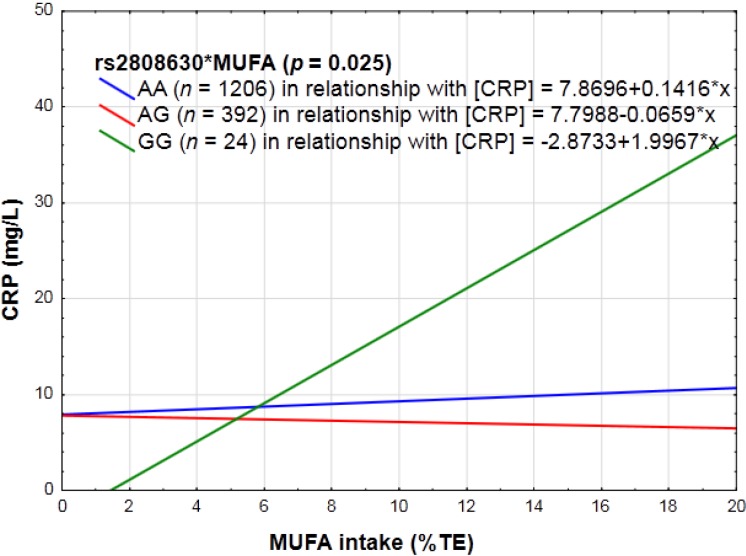
The linear relationship between monounsaturated fatty acids (MUFA) intake in relation to CRP concentrations categorized for rs2808630.

SFA and MUFA consumption, expressed as a % of TE, influenced the manner in which rs2808630 (*p* = 0.03; *p* = 0.025) affected CRP concentrations. With increasing SFA or MUFA intake, individuals homozygous for the minor allele reacted in a pro-inflammatory manner, whereas in those carrying the major allele, no effect was observed on CRP concentrations (see Figure 5). No interactive effects were observed for total fat expressed as %TE, MUFA-to-SFA ratio, PUFA expressed as %TE, omega-6, or omega-3 PUFA intake with any of the *CRP* SNPs.

**Figure 6:**
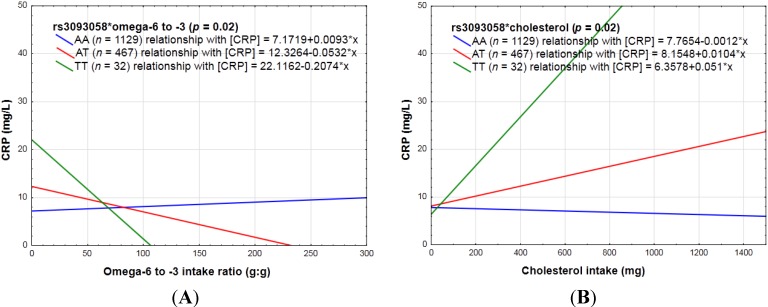
The linear relationship between (**A**) omega-6 to -3 ratio and (**B**) cholesterol in relation to CRP concentrations categorized for rs3093058.

Omega-6 to -3 ratio interacted with rs3093058 (*p* = 0.02) and rs3093062 (*p* = 0.03). When harboring the minor allele of both SNPs, a high omega-6 to -3 ratio seems to be anti-inflammatory (see Figure 6A).

Cholesterol intake interacted with rs3093058 (*p* = 0.02) and rs3093062 (*p* = 0.01), *i.e.*, with increasing cholesterol intake CRP concentrations increased in those with the minor allele in a genotype dose-dependent manner and decreased in non-carriers (see Figure 6B).

No interaction effects were observed for any of the determined *CRP* SNPs with alcohol, fiber (insoluble or soluble), or the micronutrients (vitamin C and E, zinc and magnesium). Furthermore, no interaction effects were observed for carotenoid, fruit and vegetables, pulses, nut and seed intake or any of the diet quality scores (healthy diet indicator or Thiele dietary quality score) with any of the *CRP* genotypes in relation to the CRP phenotype.

## 4. Discussion

This study is unique in that it is the first of this magnitude to investigate the variation of inflammatory responses (measured as CRP concentrations) to certain lifestyle stimuli and their interactions/associations with several *CRP* genotypes (see supplementary Table). In addition to changing baseline CRP concentrations individually, the effects of the *CRP* genetic variations were modulated by anthropometric markers such as BMI, upper-arm and waist circumference), biochemical markers such as triglycerides, HbA1c, and fasting glucose, and dietary intake, *i.e.*, MUFA-to-SFA, omega-6 to -3 ratio, and cholesterol in relation to the inflammation phenotype. Consistent results were obtained for the SNPs found to be in LD; functional studies will be needed before it will be possible to determine which SNP was responsible for the effects observed. Little is known about the manner in which nutrients affect gene expression, but it seems plausible that nutrients can affect gene expression, thus altering either messenger ribonucleic acid and/or active protein by changing transcriptional, post-transcriptional and post-translational events [31], depending on the location of the genetic variant on the chromosome. Although the precise molecular mechanisms linking nutritional status and inflammation remain unclear, this work can begin to clarify the manner in which these contribute to the inflammatory state.

### 4.1. Anthropometry and PA with CRP Genotype Interactions in Relation to Inflammation

Interactions between several *CRP* genotypes and indices of body fatness were observed. Our findings extend the existing knowledge of the link between adipose tissue and inflammation [32,33,34]. One of the ways in which the link between obesity and inflammation may be mediated, therefore, is through genotype body fat interaction effects, probably modulating the phenotype by changes in gene expression. It has been reported in previous studies that irrespective of the genetic background, diets to promote weight loss significantly diminish CRP concentrations [9,11,35]. A decrease in adipose tissue reduces interleukin 6 (IL-6) concentrations, which in turn decreases CRP synthesis [36]. The interactive effect of the indicators of body fatness on the CRP genotypes investigated is, therefore, not surprising. However, we postulate that weight loss might be especially beneficial and effective to reduce low-grade inflammation in those harboring the minor alleles at rs1130864, rs3093058 and rs3093062.

### 4.2. Biochemical Markers and CRP Genotype Interactions in Relation to Inflammation

Our results suggest that the resultant phenotype of the genetic variations at rs3093058/rs3093062 and rs11308664 are responsive to circulating triglyceride concentrations. Carlson *et al.* [15] observed that triglyceride concentrations correlated with CRP concentrations, but found, in contrast to our results, no significant associations between *CRP* haplotypes and triglycerides in relation to the change in CRP. Ladeia *et al.* [37] attributed the positive correlation between CRP and triglyceride concentration to the unfavorable lipid profile invoking additional inflammatory activity.

HbA1c and fasting glucose interacted with several genotypes, thus altering the inflammatory response. When considered together with the results of the dietary intake factors that can affect glycemic control, these results indicate that while the direct effects of added sugar or total carbohydrate intake may be indiscernible, dietary changes affecting the glycemic milieu may have a more pronounced effect on the regulation of the inflammatory phenotype. This is not unexpected, as hyperglycemia stimulates the release of cytokines [38] and leads to the induction and secretion of acute-phase proteins by adipocytes [39]. Previous studies have determined controversial results, with some indicating increasing CRP concentrations with higher fasting glucose levels [40,41], while others produced no significant associations [33,42]. Our findings could provide the answer to these discrepancies, as some genotypes reacted in a pro-inflammatory manner while others reacted in an anti-inflammatory manner to a hyperglycemic milieu.

### 4.3. Specific Dietary Components as Well as Diet Quality Scores and CRP Genotype Interactions in Relation to Inflammation

A high ratio of omega-6 to -3 intake increased inflammation in those harboring the major allele at rs3093058 and rs3093062, whereas the ratio was associated with a greater reduction in inflammation in those harboring the variant allele. For the majority of individuals (*i*.*e*., those harboring the major alleles), omega-6 may affect the inflammatory environment unfavorably, whereas omega-3 intake may be anti-inflammatory owing to the lower production of cytokines. Because of these differential *CRP* responses, dietary advice given to the general public to increase omega-3 intake in order to have a low omega-6 to -3 ratio could, therefore, have significant public health benefits to most, but might be detrimental to a small, but significant number of individuals harboring the minor alleles. Our results add to previous studies [43,44], which determined that omega-3 PUFAs, were associated with lower CRP, although these studies did not consider interactions with genetic make-up.

With increasing cholesterol consumption, only individuals carrying the minor allele at rs3093062 or rs3093058 were adversely affected with regard to inflammation in an allele dose-dependent manner. Those homozygous for the major allele may be regarded as “low responders” to cholesterol intakes in terms of inflammation. Irrespective of genetic background, Tannock *et al.* [45] demonstrated that a high-cholesterol diet led to increases in CRP, while Pirro *et al.* [46] demonstrated that the reverse was true for low-cholesterol diets.

Since diets are complex mixtures of food and the possibility of synergy between dietary factors needs to be considered, we in addition investigated diet quality, but determined that it did not modulate any of the *CRP* genotypes’ effect on the inflammation phenotype.

## 5. Conclusions

We believe our findings to be of practical and clinical importance. Firstly, observational studies mimic the real-life situation that diet and lifestyle are complex, but might fail to identify specific anti-inflammatory compounds, especially when their individual effects on inflammation are small. Despite this limitation, our approach allowed for the detection of inflammatory effects of markers of nutrition status, including individual dietary components. Secondly, these new findings are promising because, if a reduction in CRP could lead directly to a decreased rate of recurrent CVD events and the progression of atherosclerosis [47,48] and CRP could be lowered by diet, it seems prudent to make lifestyle recommendations based on the findings of this study, especially to those with a family history of CVD. A diet plan that allows for weight loss for those with unfavorable amounts of adipose tissue that is accompanied by increased consumption of unrefined carbohydrates and decreased amounts of high-GI food, prudent SFA and MUFA intake, varying amounts of omega-3 according to genetic background and moderate amounts of cholesterol could improve CRP concentrations in those whose *CRP* genotypes are susceptible to lifestyle stimuli. However, gene-directed intervention studies need to be undertaken before this can be stated definitively. Thirdly, our results revealed that genetic differences that affect *CRP* response to lifestyle intervention may require different recommendations, especially with regard to omega-6 to -3 intake ratio, as we observed heterogeneity of responses in individuals carrying certain genotypes. This may assist in the development of personalized diets, although it would be necessary to evaluate these effects in different populations. These new diet-gene interactions are important to consider in future intervention trial protocols targeting inflammation and diseases and might aid in the development of personalized effective preventative and therapeutic strategies.

## Supplementary Files

Supplementary File 1
