# Supplementary material for: Interactions between C-Reactive Protein Genotypes with Markers of Nutritional Status in Relation to Inflammation
Source: Nutrients. 2014 Nov 11;6(11):5034–50. doi: 10.3390/nu6115034 (PMC4245578; doi:10.3390/nu6115034)
Supplement: Supplementary File 1 [file nutrients-06-05034-s001.docx]

**Supplementary Information**

**Table S1.** *CRP* Gene Polymorphism Interaction with Markers of Nutritional Status.

| Interaction | Interaction *p*-Value | Genotype | *r* | *p*-Value |
| --- | --- | --- | --- | --- |
| rs1130864 * BMI (3u1273C > T or +1444C > T) | 0.004 | GG | 0.11 | <0.0001 |
|  |  | GA | −0.01 | 0.79 |
|  |  | AA | 0.39 | 0.07 |
| rs1130864 * upper-arm circumference (3u1273C > T or +1444C > T) | 0.003 | GG | 0.05 | 0.08 |
|  |  | GA | −0.06 | 0.29 |
|  |  | AA | 0.49 | 0.02 |
| rs3093058 * waist circumference (−790A > T) | 0.04 | AA | 0.07 | 0.03 |
|  |  | AT | 0.18 | <0.0001 |
|  |  | TT | 0.37 | 0.04 |
| rs3093062 * waist circumference  (−409G > A) | 0.04 | GG | 0.07 | 0.03 |
|  |  | GA | 0.19 | <0.0001 |
|  |  | AA | 0.37 | 0.4 |
| rs3093058 * triglyceride concentrations  (−790A > T) | 0.007 | AA | 0.05 | 0.11 |
|  |  | AT | 0.02 | 0.64 |
|  |  | TT | 0.50 | 0.005 |
| rs3093062 * triglyceride concentrations  (−409G > A) | 0.007 | GG | 0.05 | 0.11 |
|  |  | GA | 0.03 | 0.52 |
|  |  | AA | 0.50 | 0.005 |
| rs1130864 * triglyceride concentrations  (3u1273C > T or +1444C > T) | 0.03 | GG | 0.10 | 0.001 |
|  |  | GA | −0.05 | 0.33 |
|  |  | AA | −0.32 | 0.15 |
| rs3093058 * HbA1c  (−790A > T) | 0.005 | AA | 0.07 | 0.015 |
|  |  | AT | 0.18 | <0.0001 |
|  |  | TT | 0.28 | 0.12 |
| rs3093068 * HbA1c  (+2911C > G) | 0.006 | CC | 0.07 | 0.095 |
|  |  | CG | 0.14 | <0.0001 |
|  |  | GG | 0.19 | 0.005 |
| rs3093062 * HbA1c  (−409G > A) | 0.004 | GG | 0.07 | 0.015 |
|  |  | GA | 0.19 | <0.001 |
|  |  | AA | 0.28 | 0.12 |
| rs3093058 * fasting glucose  (−790A > T) | 0.0007 | AA | 0.003 | 0.92 |
|  |  | AT | 0.15 | 0.001 |
|  |  | TT | 0.28 | 0.12 |

**Table S1.** *Cont.*

| Interaction | Interaction *p*-Value | Genotype | *r* | *p*-Value |
| --- | --- | --- | --- | --- |
| rs1205 * fasting glucose  (3u2131C > T or 3872A > G or +2147G > A) | 0.016 | GG | 0.10 | 0.002 |
|  |  | GA | 0.003 | 0.95 |
|  |  | AA | −0.03 | 0.80 |
| rs7553007 * fasting glucose | 0.02 | GG | 0.10 | 0.003 |
|  |  | GA | 0.004 | 0.92 |
|  |  | AA | −0.2 | 0.87 |
| rs2794520 * fasting glucose | 0.019 | GG | 0.10 | 0.002 |
|  |  | GA | 0.003 | 0.95 |
|  |  | AA | −0.02 | 0.87 |
| rs2808630 * fasting glucose  (+5237A > G) | 0.018 | AA | 0.05 | 0.07 |
|  |  | AG | 0.01 | 0.84 |
|  |  | GG | 0.39 | 0.03 |
| rs3093068 * fasting glucose  (+2911C > G) | 0.001 | CC | 0.004 | 0.92 |
|  |  | CG | 0.06 | 0.13 |
|  |  | GG | 0.21 | 0.002 |
| rs3093062 * fasting glucose  (−409G > A) | 0.0004 | GG | 0.003 | 0.92 |
|  |  | GA | 0.16 | 0.001 |
|  |  | AA | 0.28 | 0.12 |
| rs2027471 * fasting glucose | 0.02 | TT | 0.10 | 0.003 |
|  |  | TA | 0.004 | 0.92 |
|  |  | AA | −0.02 | 0.85 |
| rs1341665 * fasting glucose  (−7180C > T) | 0.02 | GG | 0.10 | 0.003 |
|  |  | GA | 0.004 | 0.92 |
|  |  | AA | −0.02 | 0.85 |
| rs2808630 * SFA consumption  expressed as a % of TE  (+5237A > G) | 0.03 | AA | 0.03 | 0.39 |
|  |  | AG | 0.001 | 0.98 |
|  |  | GG | 0.50 | 0.005 |
| rs2808630*MUFA consumption expressed as a % of TE  (+5237A > G) | 0.025 | AA | 0.03 | 0.29 |
|  |  | AG | −0.02 | 0.73 |
|  |  | GG | 0.60 | <0.0001 |
| rs3093058 * omega-6 to -3 intake ratio  (−790A > T) | 0.02 | AA | 0.02 | 0.55 |
|  |  | AT | −0.10 | 0.04 |
|  |  | TT | −0.25 | 0.19 |
| rs3093062 * omega-6 to -3 intake ratio  (−409G > A) | 0.03 | GG | 0.02 | 0.56 |
|  |  | GA | −0.10 | 0.04 |
|  |  | AA | −0.25 | 0.19 |

**Table S1.** *Cont.*

| Interaction | Interaction *p*-Value | Genotype | *r* | *p*-Value |
| --- | --- | --- | --- | --- |
| rs3093058 * cholesterol intake  (−790A > T) | 0.02 | AA | −0.02 | 0.60 |
|  |  | AT | 0.12 | 0.01 |
|  |  | TT | 0.37 | 0.05 |
| rs3093062 * cholesterol intake  (−409G > A) | 0.01 | GG | −0.02 | 0.61 |
|  |  | GA | 0.13 | 0.007 |
|  |  | AA | 0.37 | 0.05 |

* Interaction with A, adenine; C, cytosine; G, guanine; T, thymine.

© 2014 by the authors; licensee MDPI, Basel, Switzerland. This article is an open access article distributed under the terms and conditions of the Creative Commons Attribution license (http://creativecommons.org/licenses/by/4.0/).
